# Supplementary material for: An Inherited Allele Confers Prostate Cancer Progression and Drug Resistance via RFX6/HOXA10‐Orchestrated TGFβ Signaling
Source: Adv Sci (Weinh). 2024 Jun 26;11(32):2401492. doi: 10.1002/advs.202401492 (PMC11348203; doi:10.1002/advs.202401492)
Supplement: Supplementary file 1 — Supporting Information [file ADVS-11-2401492-s003.pdf]

ADVANCED SCIENCE
Open Access

Supporting Information

for *Adv. Sci.*, DOI 10.1002/advs.202401492

An Inherited Allele Confers Prostate Cancer Progression and Drug Resistance via RFX6/HOXA10-Orchestrated TGFβ Signaling

*Mengjie Zhong, Wenjie Xu, Pan Tian, Qin Zhang, Zixian Wang, Limiao Liang, Qixiang Zhang, Yuehong Yang, Ying Lu and Gong-Hong Wei**

Zhong *et al*. Extended Data Figure 1

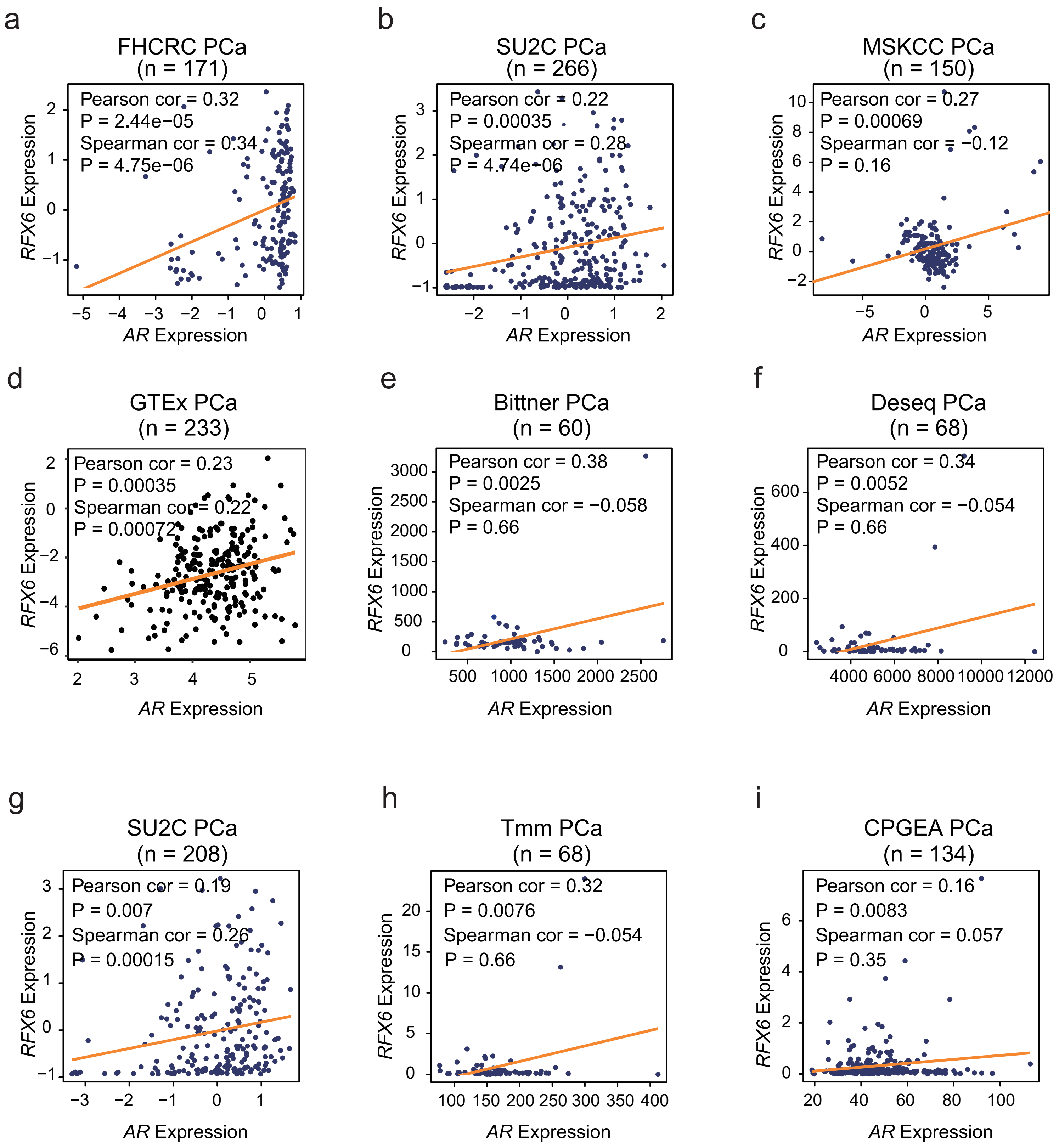

Extended Data Figure 1. Strong correlation between RFX6 and AR expression in PCa.

a-i, catter plots demonstrating a positive correlation between RFX6 and AR expression across various PCa patient cohorts, including FHCRC PCa (n = 171), SU2C PCa (n = 266, 208), MSKCC PCa (n = 150), GTEx PCa (n = 233), Bittner PCa (n = 60), Deseq PCa (n = 68), Tmm PCa (n = 68) and CPGEA PCa (n = 134) cohorts.

Statistical Analysis: Pearson and Spearman correlation analyses were employed to assess p-values across all cohorts.

Zhong et al. Extended Data Figure 2

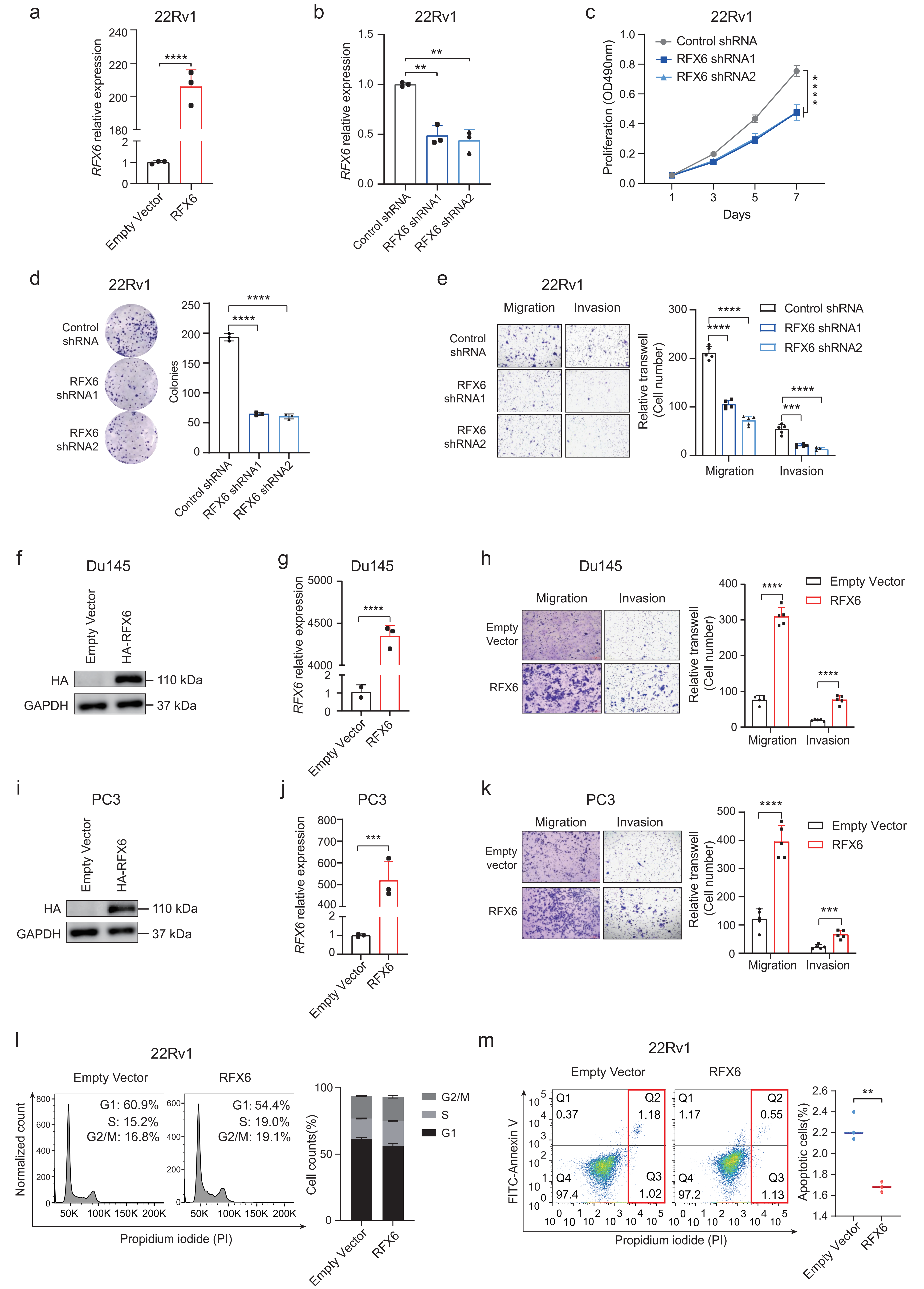

Extended Data Figure 2. Evaluating the effects of RFX6 expression alterations on PCa cell phenotype.

a-b, Analysis of RFX6 expression levels in 22Rv1 cells under various treatments, determined by quantitative real-time PCR.

c-d, Assessment of cellular proliferation in 22Rv1 cells with RFX6 knockdown using MTT assay (OD_{490}; mean ± SD from three independent experiments) (**c**) and colony formation assay (**d**).

e, Representative images and quantification of cell migration and invasion 22Rv1 cells stably expressing shRNAs targeting RFX6.

f, **i**, Immunoblots showing RFX6 protein levels in Du145 (**f**) and PC3 (**i**) cells with either empty vector or RFX6 overexpression.

g, **j**, Real-time PCR analysis of RFX6 mRNA levels normalized to GAPDH in DU145 (**g**) and PC3 (**j**) cells with varying RFX6 expression.

h, **k**, Representative images and quantification of cell migration and invasion in DU145 (**h**) or PC3 (**k**) cells with the specified RFX6 expression status.

l, Cell cycle distribution analysis of 22Rv1 cells with empty vector or RFX6 overexpression, conducted via flow cytometry, focusing on the G1, S, and G2/M phases.

m, Apoptosis assessment in 22Rv1 cells with empty vector or RFX6 overexpression using flow cytometry.

All the statistical significance assessed using the two-tailed Student's t tests. Significance levels indicated as *p < 0.05, **p < 0.01, ***p < 0.001, ****p < 0.0001.

Zhong *et al*. Extended Data Figure 3

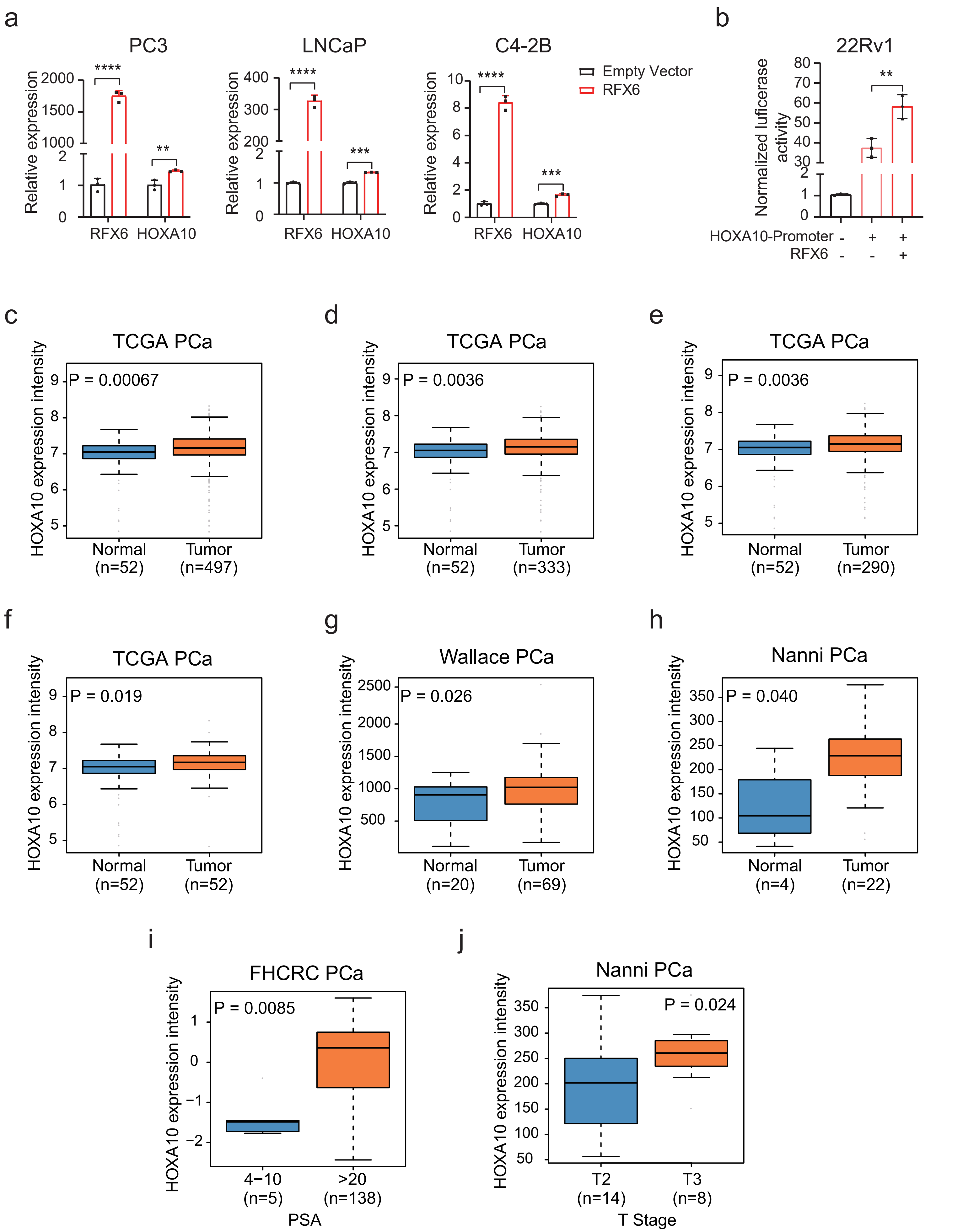

Extended Data Figure 3. RFX6 regulation of HOXA10 expression in various PCa cell lines.

a, Analysis of HOXA10 expression normalized to GAPDH in PC3, LNCaP, and C4-2B cell lines with stable RFX6 overexpression, measured by quantitative real-time PCR.

b, Luciferase reporter assay demonstrating enhanced transcriptional activity of the HOXA10 promoter in 22Rv1 cells with RFX6 overexpression compared to empty vector control.

c-h, Clinical data analyses indicating a correlation between HOXA10 upregulation and human prostate tumor progression.

i-j, Clinical data showing associations of HOXA10 upregulation with elevated levels of prostate-specific antigen (PSA) (**i**) and higher tumor stage (**j**) in PCa.

Statistical Analysis: Two-tailed Student’s t-tests were employed for all data points to assess statistical significance and p-values, with significance levels denoted as *$p < 0.05$, **$p < 0.01$, ***$p < 0.001$, ****$p < 0.0001$.

Zhong *et al*. Extended Data Figure 4

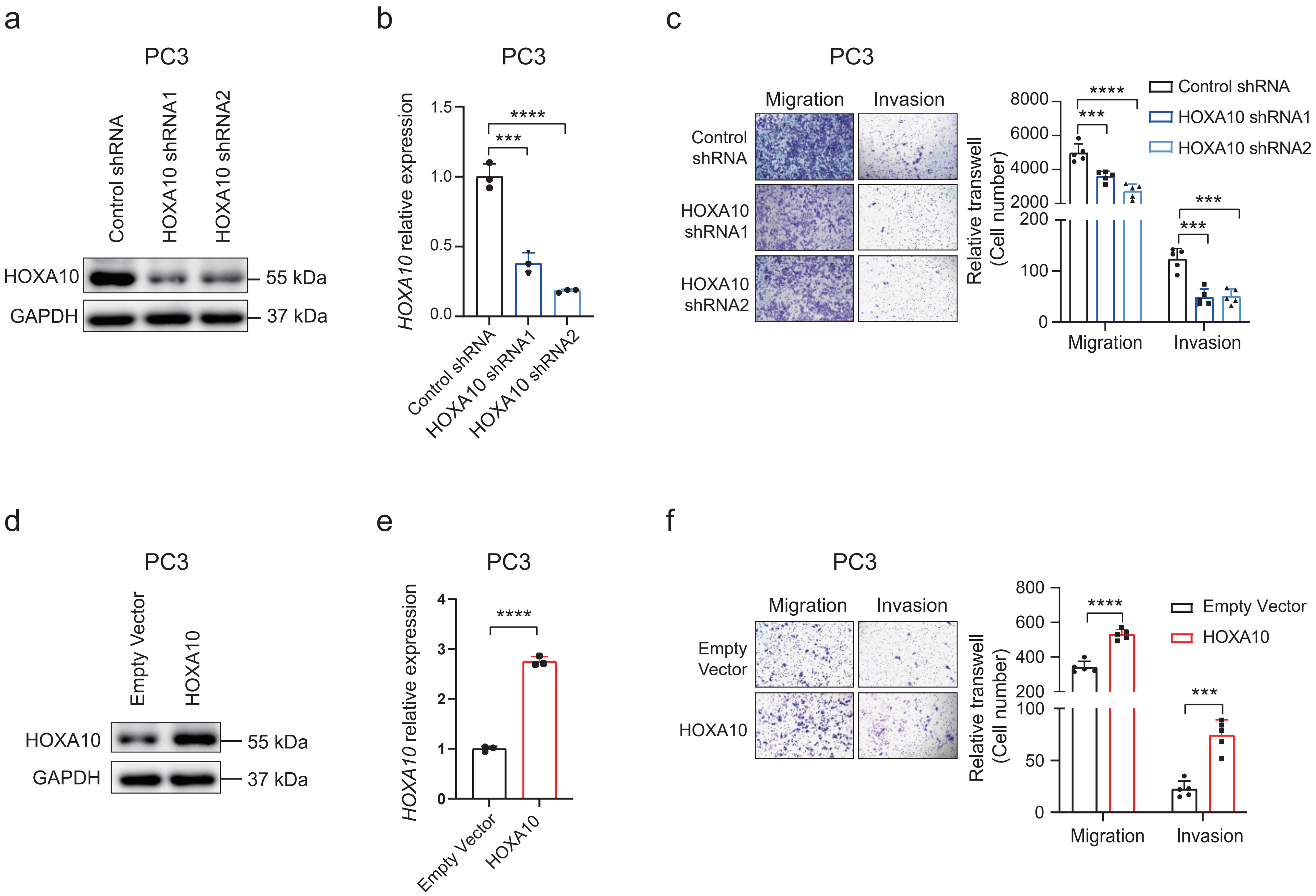

Extended Data Figure 4. Investigating the effects of HOXA10 expression alterations on PC3 cell phenotype.

a-b, Verification of shRNA-mediated RFX6 knockdown efficiency in PC3 cells, assessed by immunoblotting (**a**) and quantitative real-time PCR (**b**).

c, Representative images showcasing migration and invasion of PC3 cells stably expressing shRNAs targeting RFX6.

d, Immunoblots illustrating HOXA10 protein levels in PC3 cells with either empty vector or HOXA10 overexpression.

e, Quantitative real-time PCR analysis of relative HOXA10 mRNA expression levels normalized to GAPDH in PC3 cells with empty vector or HOXA10 overexpression.

f, Representative images of migration and invasion assays in PC3 cells transfected with either empty vector or HOXA10 expression construct.

Statistical Analysis: All data were analyzed for statistical significance using two-tailed Student’s t-tests. Significance levels are indicated as $^{*}p < 0.05$, $^{**}p < 0.01$, $^{***}p < 0.001$, $^{****}p < 0.0001$.

Zhong *et al*. Extended Data Figure 5

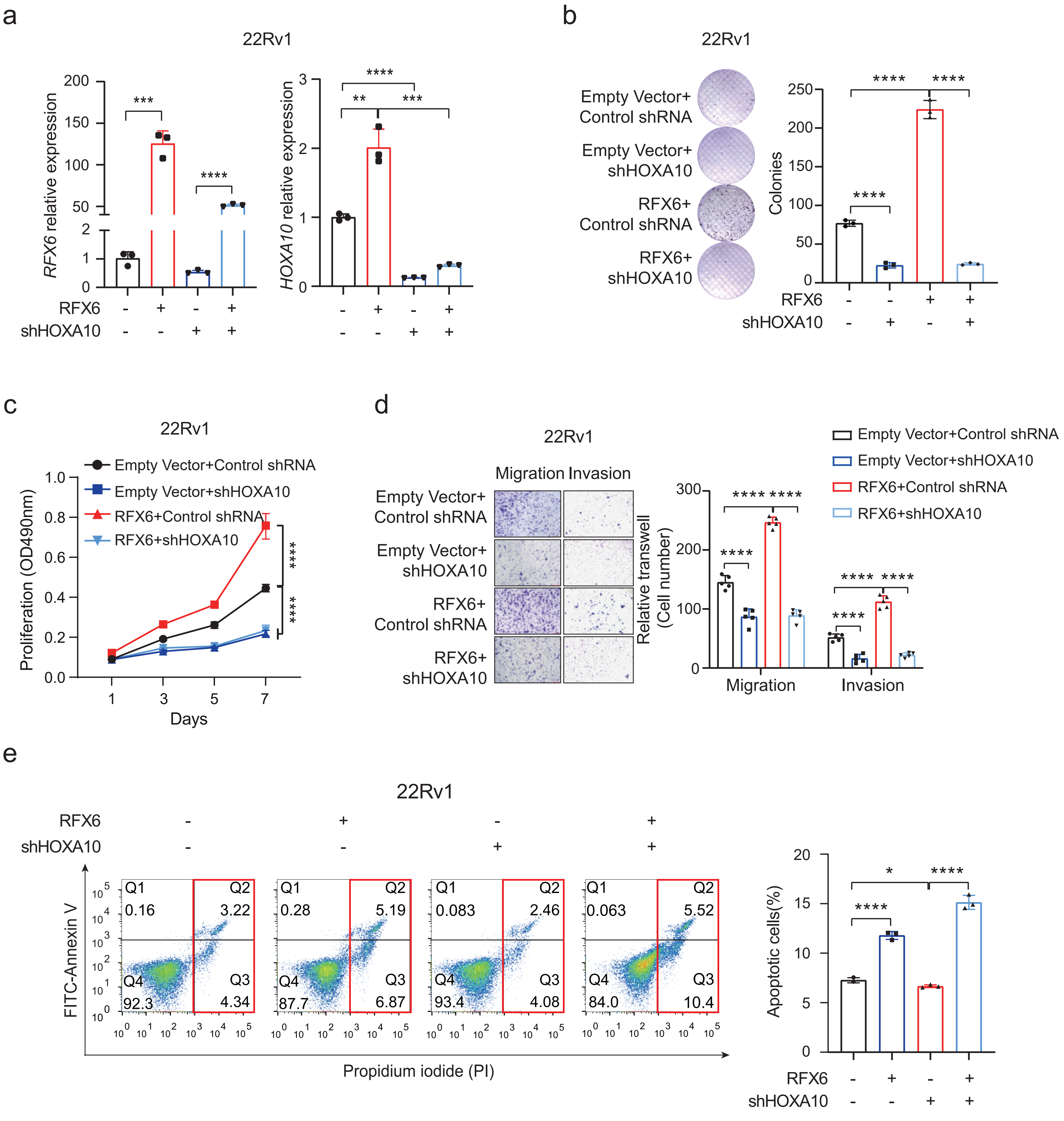

Extended Data Figure 5. Reversal of RFX6-induced cellular phenotype by HOXA10 suppression *in vitro*.

a, Analysis of relative expression levels of RFX6 and HOXA10 in 22Rv1 cells under various treatments, measured by quantitative real-time PCR.

b-c, Evaluation of cellular proliferation in 22Rv1 cells with the specified treatments using colony formation assay (**b**) and MTT assay (OD_{490}; mean ± SD from three independent experiments) (**c**).

d, Representative images and analysis of cell migration and invasion in 22Rv1 cells stably expressing different treatment groups.

e, Flow cytometry analysis to determine the proportion of apoptotic cells in the indicated treatment groups.

Statistical Analysis: Statistical significance for all data points was determined using two-tailed Student's t-tests. Significance levels are denoted as $*p < 0.05$, $**p < 0.01$, $***p < 0.001$, $****p < 0.0001$.

Zhong *et al*. Extended Data Figure 6

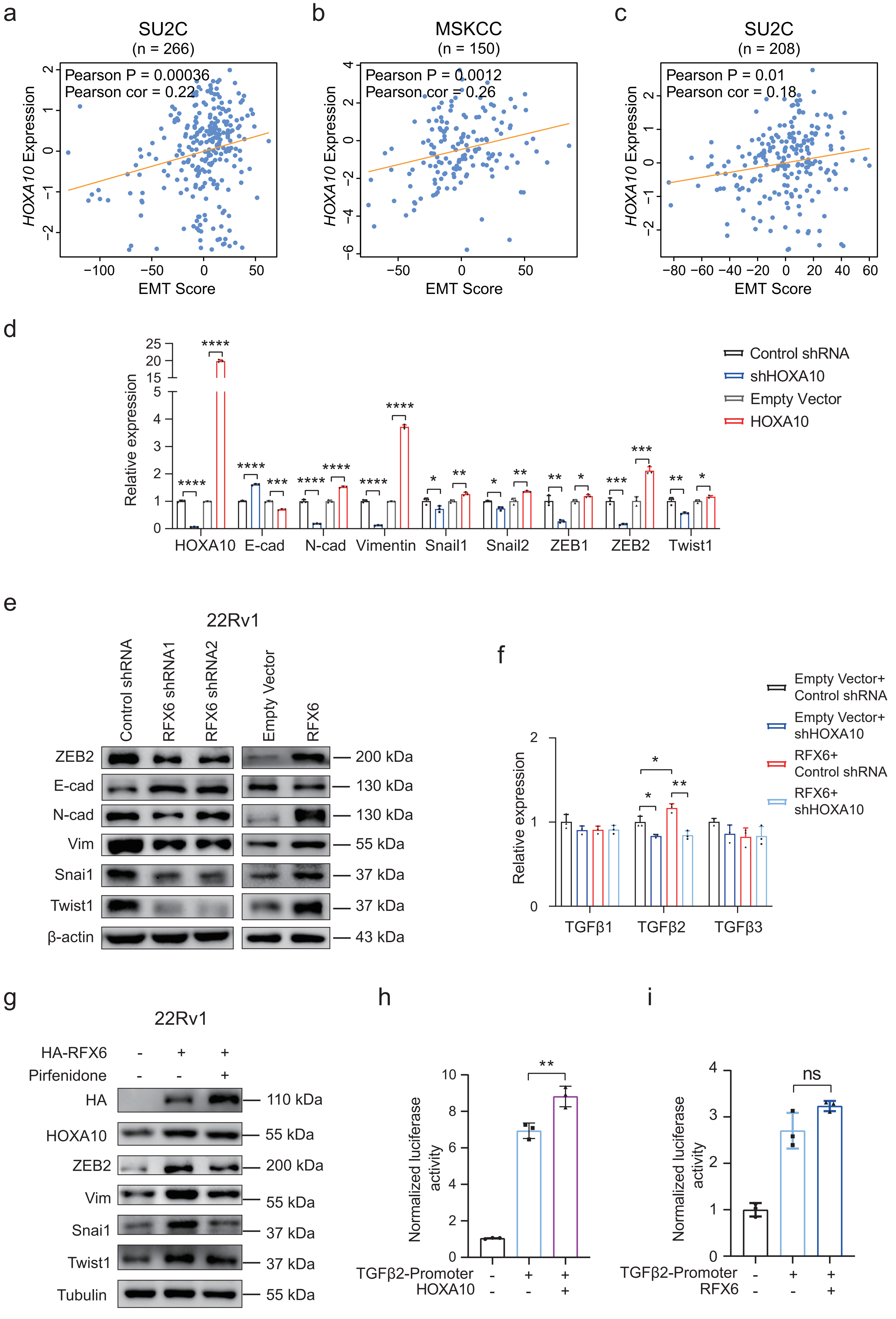

Extended Data Figure 6. RFX6 and HOXA10 expression impact on EMT marker expression via TGFβ2 modulation.

a-c, Positive correlation between HOXA10 expression and computed EMT scores in various independent PCa cohorts.

d, Real-time PCR analysis of EMT-related molecules (E-cadherin, N-cadherin, Vimentin, Snail, Slug, ZEB1, ZEB2, Twist1) in cells with specified treatments.

e, Immunoblotting determination of EMT-related molecules (E-cadherin, N-cadherin, Vimentin, Snail1, ZEB2, Twist1) in cells under indicated treatment conditions.

f, Quantitative analysis of TGFβ1, TGFβ2, and TGFβ3 mRNA levels in 22Rv1 cells with various treatments.

g, Assessment of EMT-associated molecules (E-cadherin, N-cadherin, vimentin, Snail, ZEB2, Twist1) in 22Rv1 cells following Pirfenidone treatment, using immunoblotting.

h-i, Luciferase reporter assays evaluating the impact of HOXA10 (**h**) and RFX6 (**i**) on the transcriptional activity of the TGFβ2 promoter in 22Rv1 cells, compared to the empty vector control.

Statistical Analysis: All data were analyzed for statistical significance using two-tailed Student's t-tests. Significance levels are indicated as $*p < 0.05$, $**p < 0.01$, $***p < 0.001$, $****p < 0.0001$.

Zhong *et al*. Extended Data Figure 7

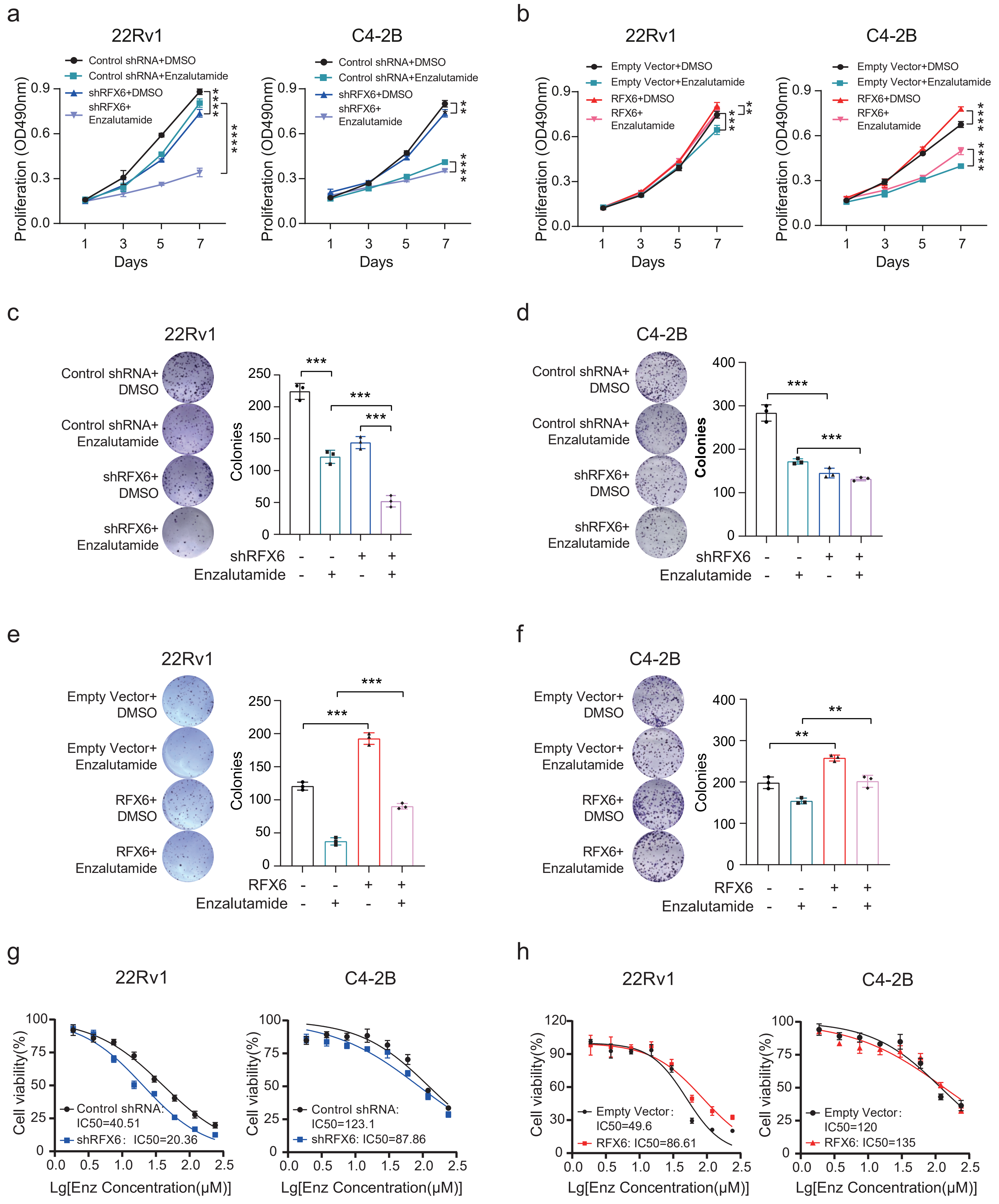

Extended Data Figure 7. Effects of RFX6 levels on enzalutamide sensitivity in CRPC cell lines.

a-f, Evaluation of cellular proliferation in 22Rv1 or C4-2B cells under various treatments, measured using MTT assay (OD_{490}; mean ± SD from three independent experiments) (**a-b**) and colony formation assay (**c-f**).

g-h, Determination of the half-maximal inhibitory concentration (IC50) of enzalutamide in 22Rv1 or C4-2B cells, based on the indicated treatments.

Statistical Analysis: Two-tailed Student's t-tests were utilized to assess statistical significance across all data points. Significance levels are denoted as *p < 0.05, **p < 0.01, ***p < 0.001, ****p < 0.0001.
